# Single Nucleotide Polymorphisms in Regulator-Encoding Genes Have an Additive Effect on Virulence Gene Expression in a *Vibrio cholerae* Clinical Isolate

**DOI:** 10.1128/mSphere.00253-16

**Published:** 2016-09-21

**Authors:** Bailey M. Carignan, Kyle D. Brumfield, Mike S. Son

**Affiliations:** Department of Biological Sciences, Plymouth State University, Plymouth, New Hampshire, USA; University of Michigan

**Keywords:** Toxin-coregulated pilus, *Vibrio cholerae*, cholera toxin, *hapR*, *hns*, *luxO*, *toxT*, *vieA*, virulence factors

## Abstract

*Vibrio cholerae* is the etiological agent of the infectious disease cholera, which is characterized by vomiting and severe watery diarrhea. Recently, *V. cholerae* clinical isolates have demonstrated increased virulence capabilities, causing more severe symptoms with a much higher rate of disease progression than previously observed. We have identified single nucleotide polymorphisms (SNPs) in four virulence-regulatory genes (*hapR*, *hns*, *luxO*, and *vieA*) of a hypervirulent *V. cholerae* clinical isolate, MQ1795. Herein, all SNPs and SNP combinations of interest were introduced into the prototypical El Tor reference strain N16961, and the effects on the production of numerous virulence-related factors, including cholera toxin (CT), the toxin-coregulated pilus (TCP), and ToxT, were analyzed. Our data show that triple-SNP (*hapR hns luxO* and *hns luxO vieA*) and quadruple-SNP combinations produced the greatest increases in CT, TCP, and ToxT production. The *hns* and *hns luxO* SNP combinations were sufficient for increased TCP and ToxT production. Notably, the *hns luxO vieA* triple-SNP combination strain produced TCP and ToxT levels similar to those of MQ1795. Certain SNP combinations (*hapR* and *hapR vieA*) had the opposite effect on CT, TCP, and ToxT expression. Interestingly, the *hns vieA* double-SNP combination strain increased TCP production while decreasing CT production. Our findings suggest that SNPs identified in the four regulatory genes, in various combinations, are associated with increased virulence capabilities observed in *V. cholerae* clinical isolates. These studies provide insight into the evolution of highly virulent strains.

**IMPORTANCE** Cholera, an infectious disease of the small intestine caused by the aquatic bacterium *Vibrio cholerae*, often results in vomiting and acute watery diarrhea. If left untreated or if the response is too slow, the symptoms can quickly lead to extreme dehydration and ultimately death of the patient. Recent anecdotal evidence of cholera patients suffering from increasingly severe symptoms and of disease progression at a much higher rate than previously observed has emerged. As recent cholera outbreaks caused by increasingly virulent strains have resulted in higher mortality rates, the need to investigate the mechanism(s) allowing this observed increased virulence is apparent. The significance of our research is in identifying the mechanism for increased virulence capabilities, which will allow the development of a model that will greatly enhance our understanding of cholera disease and *V. cholerae* pathogenesis, leading to broader biomedical impacts, as cholera serves as a model for other enteric diarrheal diseases.

## Abstract (precis)

Cholera, an infectious disease of the small intestine caused by the aquatic bacterium *Vibrio cholerae*, often results in vomiting and acute watery diarrhea. If left untreated or if the response is too slow, the symptoms can quickly lead to extreme dehydration and ultimately death of the patient. Recent anecdotal evidence of cholera patients suffering from increasingly severe symptoms and of disease progression at a much higher rate than previously observed has emerged. As recent cholera outbreaks caused by increasingly virulent strains have resulted in higher mortality rates, the need to investigate the mechanism(s) allowing this observed increased virulence is apparent. The significance of our research is in identifying the mechanism for increased virulence capabilities, which will allow the development of a model that will greatly enhance our understanding of cholera disease and *V. cholerae* pathogenesis, leading to broader biomedical impacts, as cholera serves as a model for other enteric diarrheal diseases.

## INTRODUCTION

Globally, cholera is a reemerging infectious disease of the lower intestines caused by the bacterium *Vibrio cholerae* and is characterized by severe diarrhea and dehydration. *V. cholerae* has been categorized into over 200 serogroups, of which only two, O139 and O1, have been shown to have epidemic or pandemic potential (1, 2). Serogroup O139 was previously isolated from patients with mild diarrhea, primarily in Southeast Asia (3, 4). However, serogroup O1 can be found worldwide and is responsible for all current and previously reported pandemics since 1817.

The toxin-coregulated pilus (TCP) and cholera toxin (CT) are two main virulence factors produced by *V. cholerae* O1, which allows the bacterium to colonize and establish an infection in a host and to cause the physical symptoms of the disease, respectively. Increased expression of the TCP, a type IV pilus expressed by the *tcp* operon (*tcpABQCRDSTEF*) located on the *Vibrio* pathogenicity island (VPI), has been associated with enhanced attachment (5) and is essential for colonization of the intestinal epithelium (6–8). The first gene in the *tcp* operon, *tcpA*, codes for the individual pilin subunits that comprise the pilus structure (8) and can be used to quantitate overall TCP production (9). Additionally, colonization of the intestinal epithelium, mediated through the TCP, is critical for the subsequent delivery of CT *in vivo* (6, 7, 10). CT, encoded by the *ctxAB* operon located on the CTXφ prophage, is a bipartite toxin responsible for inducing severe watery diarrhea and the subsequent electrolyte loss associated with cholera (11). The ultimate expression of these two virulence factors, TCP and CT, is regulated by ToxT, the master virulence regulator, through a cascade of transcriptional regulators. Briefly, accessory proteins AphA and AphB regulate the expression of TcpPH (12–14). TcpPH, in cooperation with ToxRS, then allows the expression of *toxT* (15–17).

Serogroup O1 can be subdivided into two biotypes, classical and El Tor, which individually display unique genotypic and phenotypic traits (3, 9, 18, 19). The classical biotype, which is responsible for the first six cholera pandemics, produces higher levels of CT and causes more severe disease than the El Tor biotype (20). The current (seventh) pandemic (1961 to the present) is caused by the El Tor biotype, which displaced the classical biotype in the environment beginning around 1993 (21–23). Between both biotypes, *ctxA* is completely conserved, while *ctxB* and *tcpA*, although conserved within biotypes, differ across the two biotypes, allowing for reliable biotype characterization (9).

This distinction serves as a primary focus of epidemiological studies, but evidence indicating that El Tor biotype strains isolated as far back as the early 1990s possessed various classical biotype traits has begun to emerge (9, 20, 24–30). These were later termed El Tor variants (27, 31). One particular El Tor variant, MQ1795, was reported to produce more CT than El Tor and classical reference strains and was designated hypervirulent following increased virulence observed in an infant mouse cholera model (9).

Although the mechanism by which *V. cholerae* establishes infection has been well characterized (6–8), the underlying mechanisms behind increased virulence observed in some *V. cholerae* clinical isolates are still under investigation (9, 20). Previously, single nucleotide polymorphisms (SNPs) were identified through next-generation deep sequencing (9) in four regulatory genes (*hapR*, *hns*, *luxO*, and *vieA*). These four SNPs were identified in various combinations across 11 El Tor variant clinical isolates. To date, no specific SNP(s) or SNP combination(s) can account for the increased virulence observed across the clinical isolates; however, SNPs in all four regulatory genes (Table 1) were previously reported in the hypervirulent MQ1795 strain (9). Each of the four regulatory genes has been shown to play a vital role in governing virulence gene expression (32–35), and their roles are briefly described here.

**TABLE 1:** SNPs previously identified in MQ1795 by next-generation deep sequencing^a^

Gene	Gene identifier	Base no.	N16961		MQ1795	
			Base	Residue	Base	Residue
*hapR*	VC0583	219	None (T deletion)	Nonfunctioning	T (insertion)	Functioning
*hns*	VC1130	319	G	G	A	S
*luxO*	VC1021	656	C	A	T	V
*vieA*	VC1652	235	C	L	T	F

^a^ Single nucleotide polymorphisms (SNPs) of interest found in the hypervirulent MQ1795 strain compared to the El Tor reference strain N16961. MQ1795 has a gain of function in the *hapR* gene with a mutation that results in the insertion of a thymine. The remaining SNPs (*hns*, *luxO*, and *vieA*) result in neither a gain of function nor a loss of function but rather result in residue changes.

The gene *hns* encodes the histone-like nucleoid structuring protein, H-NS, which is a promiscuous promoter binding protein. In *V. cholerae*, H-NS has been shown to downregulate multiple levels in the virulence cascade through a mechanism of transcriptional silencing by directly binding to promoters in the *ctxAB* operon, the *tcp* operon, and the promoter region for *toxT* (33).

The gene *hapR* encodes the quorum-sensing regulatory protein HapR, which directly binds to various promoters and regulates gene/operon expression (32). At high cell densities, functional HapR binds to the *aphA* promoter, downregulating *tcpPH* expression and thus downregulating the virulence cascade (32). Interestingly, in some pathogenic strains of *V. cholerae*, such as classical O395 and El Tor N16961, this density-dependent control of virulence gene expression is lost due to a naturally occurring frameshift in the *hapR* gene (32). Despite the adverse effect of functional HapR on virulence gene expression, previous studies indicate that the percentage of *V. cholerae* strains containing functional quorum-sensing systems regulated by HapR is higher in toxigenic strains than in nontoxigenic strains (36).

LuxO, encoded by the gene *luxO*, is a quorum-sensing regulator that works in a manner contrary to that of HapR (34). At low cell densities, high levels of phosphorylated LuxO (P-LuxO) inhibit *hapR* expression by activating four small RNAs that destabilize *hapR* mRNA, indirectly upregulating the virulence cascade (32, 34, 37).

The response regulator VieA, encoded by *vieA*, is part of a three-component signal transduction system involved in enhancing CT production (35, 38). VieA contains a domain that binds and degrades the prokaryotic secondary-messenger cyclic diguanylate monophosphate (c-diGMP). High intracellular levels of c-diGMP inhibit the expression of both the *ctxAB* operon and *toxT*, and degradation of this molecule by VieA is necessary to restore wild-type (WT) CT and ToxT levels (35, 38, 39).

Herein, we describe the introduction of all aforementioned SNPs and SNP combinations identified in hypervirulent MQ1795 into the El Tor reference strain N16961 to investigate the effects that these SNPs have on the master regulator of virulence gene expression, ToxT, and the two main virulence factors, CT and TcpA. We hypothesized that the introduction of these SNPs affects the expression of the master regulator and the two main virulence factors and that a minimum SNP combination is required to recapitulate virulence factor expression observed in hypervirulent MQ1795. Our findings indicate that two triple-SNP combinations (*hapR hns luxO* and *hns luxO vieA*) and the quadruple-SNP combination strains produced the greatest increases in CT, TcpA, and ToxT production. The *hns* and *hns luxO* SNP combination strains increased only TcpA and ToxT production. Notably, the *hns luxO vieA* SNP combination strain produced levels of TcpA and ToxT that are comparable to those of the hypervirulent MQ1795 strain. Some SNP combinations (*hapR* and *hapR vieA*) led to decreased CT, TcpA, and ToxT expression. When CT and TcpA production was evaluated, similar patterns were revealed for SNPs affecting ToxT production, except the *hns vieA* SNP combination; a strain with this combination exhibited no statistically significant change in ToxT production and yet increased TcpA and decreased CT production. These data demonstrate that the regulatory mechanism(s) behind increased virulence capabilities observed in clinical isolates of *V. cholerae* are far more intricate than previously reported. Additionally, the work presented here may further the understanding of each gene’s individual role in regulating virulence gene expression.

## RESULTS

### Production profiles revealed the greatest fold increase in CT in the *hapR hns luxO vieA* SNP combination strain relative to the CT level in N16961.

**FIG 1:**
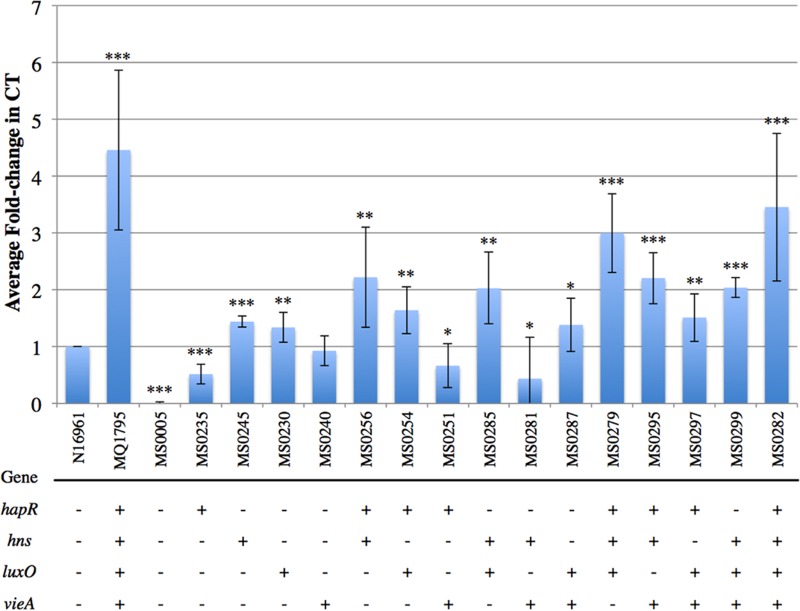
CT production profiles of single-SNP strains and double-, triple-, and quadruple-SNP combination strains. SNPs and SNP combinations were introduced into the WT strain N16961, and average fold changes in CT production levels relative to the CT production in WT N16961 were calculated. Other controls included MQ1795 (hypervirulent clinical isolate) and O395Δ*toxT* (negative control). Strains containing the different SNPs and SNP combinations introduced into WT N16961 are indicated by the numbers beginning with “MS” found in Table 2. The presence of SNPs identified in genes in the indicated clinical isolates is denoted by a “+,” whereas the WT N16961 version of a SNP is denoted by a “−.” A two-tailed Student *t* test yielded *P* values of ≤0.05 (*), <0.005 (**), and <0.0005 (***).

CT assays revealed that the MQ1795 clinical isolate produced significantly higher levels, ~4.5-fold, of CT than N16961, a well-studied El Tor strain (Fig. 1). To assess the effects of the SNPs on CT production in N16961, assays were performed using the cell-free supernatants of strains with separate SNP combinations to determine the amount of CT produced relative to that produced by the parental strain (N16961). O395Δ*toxT*, used as a negative control, produced negligible CT, as expected.

Single-SNP strains (Fig. 1) produced slight but significant changes in levels of CT, with fold increases observed in the *hns* and *luxO* single-SNP strains (1.4- and 1.3-fold). The *hapR* single-SNP strain exhibited decreased CT production relative to N16961 (0.5-fold). Double-SNP combinations (Fig. 1) produced more CT than the single-SNP strains. The *hapR hns*, *hapR luxO*, *hns luxO*, and *luxO vieA* SNP combination strains revealed increases in CT production compared to N16961 of 2.2-, 1.6-, 2.0-, and 1.4-fold, respectively. Decreases in CT were also observed in the double-SNP combinations, as the *hapR vieA* and *hns vieA* SNP combination strains produced decreases of 0.7- and 0.4-fold, similar to that of the *hapR* single-SNP strain (0.5-fold). All triple- and quadruple-SNP combinations demonstrated elevated levels of CT production. The quadruple-SNP combination increased CT production 3.5-fold relative to that of N16961, closely mirroring the level of the hypervirulent MQ1795 strain. The *hapR hns luxO*, *hapR hns vieA*, *hapR luxO vieA*, and *hns luxO vieA* SNP combination strains also yielded increases in CT of 3.0-, 2.2-, 1.5-, and 2.0-fold, respectively.

### TcpA production profiles varied across the SNP combination strains.

**FIG 2:**
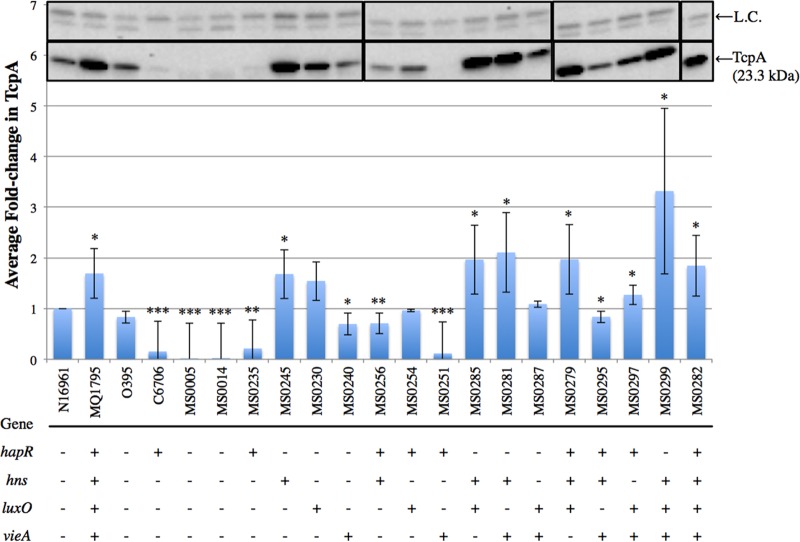
TcpA production profiles of single-SNP strains and double-, triple-, and quadruple-SNP combination strains. WCE were prepared, 3 µg of total protein was loaded onto a 16% Tris-glycine polyacrylamide gel, and proteins in immunoblots were quantified using densitometry for *V. cholerae* virulence factor TcpA. Immunoblots (inset) are from different blots (indicated by borders), all with WT N16961 and other controls run simultaneously. Nonspecific bands serve as a loading control (L.C.) and are labeled accordingly. Average fold changes were calculated relative to the TcpA level in N16961. Other controls included MQ1795 (hypervirulent clinical isolate), classical O395, El Tor C6706, O395Δ*toxT*, and O395Δ*tcpA*. Strains containing the different SNPs and SNP combinations introduced into WT N16961 are indicated by the numbers beginning with “MS” found in Table 2. The presence of SNPs identified in genes in the indicated clinical isolates is denoted by a “+,” whereas the WT N16961 version of a SNP is denoted by a “−.” A two-tailed Student *t* test yielded *P* values of ≤0.05 (*), <0.005 (**), and <0.0005 (***).

To determine TcpA (23.5 kDa) production levels relative to that of the El Tor reference strain N16961, all strains were grown under AKI virulence gene-inducing conditions, and then whole-cell extracts (WCE) for all strains were prepared. TcpA production profiles (Fig. 2) revealed that MQ1795 produced 1.7-fold-more TcpA than the reference strain N16961. Classical O395 produced amounts of TcpA similar to that of N16961, and TcpA production was greatly reduced or abolished in the classical O395Δ*toxT* and O395Δ*tcpA* backgrounds. C6706, an additional El Tor reference strain, demonstrated decreased TcpA production compared to that of N16961.

The single-SNP *hapR* strain decreased TcpA production to 0.2-fold that of N16961, while the other single-SNP strains showed no significant change in TcpA production (Fig. 2). Double-SNP combinations had various effects on TcpA production. Decreases in TcpA production were observed in the *hapR hns* and *hapR vieA* SNP combination strains, which produced 0.7- and 0.1-fold the level produced by N16961, similar to the production profile observed in the *hapR* single-SNP strain. The *hns vieA* SNP combination strain demonstrated increased TcpA production, i.e., up to 2.1-fold that of N16961, slightly higher than the level of TcpA produced by the hypervirulent MQ1795 strain. Additionally, the *hapR hns luxO* and *hns luxO vieA* triple-SNP combination strains and the *hapR hns luxO vieA* quadruple-SNP combination strain demonstrated increased levels of TcpA production (2.0-, 3.3-, and 1.8-fold, respectively), all greater than that of MQ1795 (Fig. 2).

### ToxT production profiles vary across the SNP combination strains.

**FIG 3:**
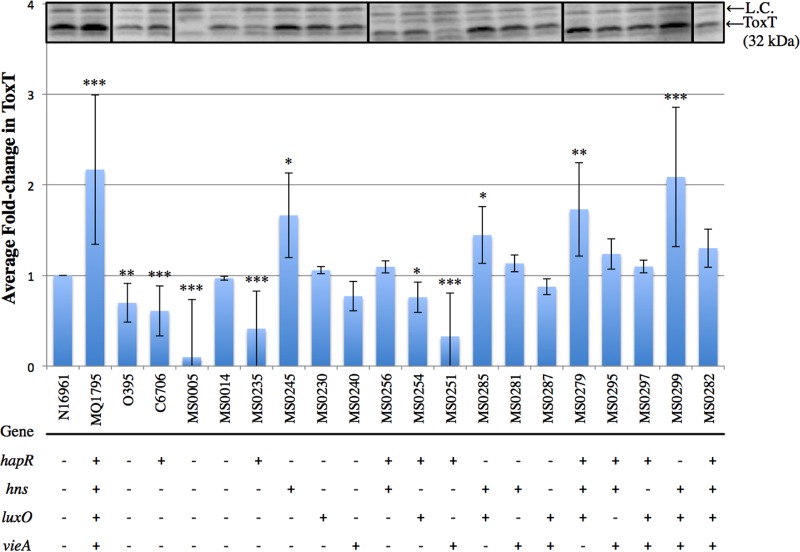
ToxT production profiles of single-SNP strains and double-, triple-, and quadruple-SNP combination strains. WCE were prepared, 12 µg of total protein was loaded onto a 14% Tris-glycine polyacrylamide gel, and proteins in immunoblots were quantified using densitometry for *V. cholerae* virulence factor ToxT. Immunoblots (inset) are from different blots (indicated by borders), all with WT N16961 and other controls run simultaneously. Nonspecific bands serve as a loading control (L.C.) and are labeled accordingly. Average fold changes were calculated relative to the ToxT level in N16961. Other controls included MQ1795 (hypervirulent clinical isolate), classical O395, El Tor C6706, O395Δ*toxT*, and O395Δ*tcpA*. Strains containing the different SNPs and SNP combinations introduced into WT N16961 are indicated by the numbers beginning with “MS” found in Table 2. The presence of SNPs identified in genes in the indicated clinical isolates is denoted by a “+,” whereas the WT N16961 version of a SNP is denoted by a “−.” A two-tailed Student *t* test yielded *P* values of ≤0.05 (*), <0.005 (**), and <0.0005 (***).

After strains were grown under AKI virulence gene-inducing conditions, the WCE for all strains were prepared to determine the ToxT (32 kDa) production levels relative to that of the El Tor reference strain N16961. ToxT production profiles (Fig. 3) revealed that MQ1795 produced 2.2-fold-more ToxT than the reference strain N1691. Classical O395 and El Tor C6706 produced similar ToxT levels, both less than that of N16961 (0.7- and 0.6-fold, respectively). As expected, O395Δ*tcpA* showed no detectable change in ToxT production relative to N16961, and O395Δ*toxT* produced no detectable ToxT and was used as a negative control.

The *hapR* single-SNP strain decreased ToxT production to 0.4-fold that of N16961. *hapR luxO* and *hapR vieA* double-SNP combination strains also presented decreases in ToxT production to 0.8- and 0.3-fold that of N16961. Conversely, the *hns luxO* strain and *hapR hns luxO* and *hns luxO vieA* triple-SNP combination strains demonstrated increased ToxT production levels of up to 1.4-, 1.7-, and 2.1-fold that of N16961 (Fig. 3). The greatest increase in ToxT production was observed with the *hns luxO vieA* triple-SNP combination strain, which increased ToxT production up to 2.1-fold that of N16961, similar to the ToxT production level observed in hypervirulent MQ1795.

## DISCUSSION

**FIG 4:**
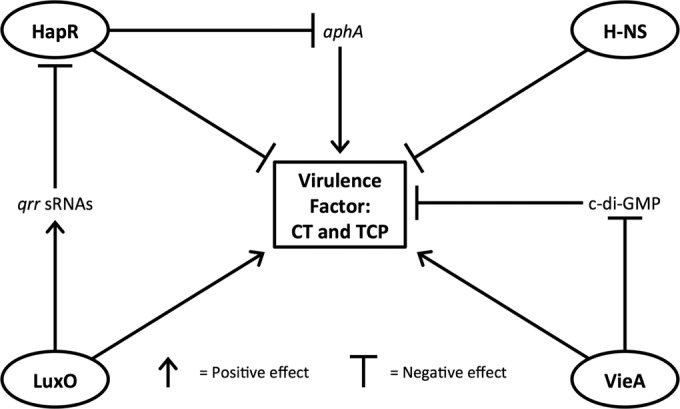
Schematic representation of the involvement of each of the four genes on virulence factor expression. HapR binds to the *aphA* promoter, resulting in a decrease in virulence factor production, and H-NS decreases virulence factor production through a mechanism of transcriptional silencing. LuxO works in a manner contrary to that of HapR, and VieA degrades c-di-GMP, both of which result in indirect upregulation of virulence factor production. qrr, quorum regulatory RNA; sRNA, small RNA.

In this study, we determined the effects of clinically relevant SNPs identified in four regulatory genes (*hapR*, *hns*, *luxO*, and *vieA*) on the virulence capabilities of *V. cholerae*. We introduced all possible SNP combinations into the El Tor reference strain N16961 and observed their effects on CT, ToxT, and TcpA production levels relative to those of N16961. We have provided a model (Fig. 4) illustrating the expected effect that each of the four genes has on virulence gene expression; however, our data proposes a far more complex mechanism of virulence regulation and interactions than previously reported.

It was previously reported that H-NS binding downregulates multiple steps in the ToxR virulence cascade (33, 40). However, when the nonsynonymous SNP in *hns* (G_319_A, residue G_107_S) was introduced into N16961, increases in CT (1.4-fold), TcpA (1.7-fold), and ToxT (1.7-fold) production (Fig. 1) were observed, suggesting that this SNP decreases H-NS activity. H-NS has previously demonstrated the greatest repressive effect on the promoter regions of *toxT* and the *ctxAB* operon and has also been shown to produce a modest repressive effect on the *tcp* operon (33). These data indicate that the SNP in *hns* produces the greatest increases in the production of TcpA and ToxT. Notably, all SNP combination strains exhibiting statistically significant increases in ToxT production contained the *hns* SNP, and all strains exhibiting a fold change greater than 1.5-fold across all assays contained the SNP in *hns*, with the exception of the *hapR luxO* double-SNP combination strain, which increased CT production to 1.6-fold that of N16961. Previously, *hns* mutations have been shown to result in derepressed expression of CT and TCP under nonpermissive and permissive ToxR conditions (33). These findings are consistent with our data, which show increases across CT, TCP, and ToxT production (Fig. 1 to 3) under ToxR-permissive conditions.

The SNP identified in *hapR* is a thymine insertion in the coding region at base 219, which results in a gain of function through the production of functional HapR. HapR is a promiscuous promoter binding protein involved in downregulating the virulence cascade by binding to the *aphA* promoter, resulting in decreased *tcpPH* expression (32). The production of functional HapR in N16961 decreased production of CT (0.5-fold) (Fig. 1), TcpA (0.2-fold) (Fig. 2), and ToxT (0.2-fold) (Fig. 3). However, HapR does not always result in decreased virulence capabilities. For example, the addition of functional *hapR* to *hns luxO vieA* in the quadruple-SNP combination strain further increased CT production relative to that of the *hns luxO vieA* triple-SNP combination strain (from 2- to 3.5-fold) (Fig. 1).

LuxO has been well characterized for its role in quorum sensing (34, 41) (for reviews, see references 42 and 43). The regulatory activity of LuxO has been proposed to be linked in a contrary manner to HapR, where at low cell densities, high levels of phosphorylated LuxO reduce the accumulation of the HapR message (32, 34, 37). This reduced level of HapR message, and therefore of HapR, results in increased expression of the virulence cascade beginning at the expression of *tcpPH*, as there is less HapR to bind to the *aphA* promoter upstream of the ToxT regulatory cascade. The *luxO* single SNP (C_656_T, residue A_219_V) resulted in increased CT production (1.3-fold); however, no statistically significant changes in TcpA or ToxT production were observed. We hypothesized an increase in ToxT production at low cell densities (3.5 h) in the *hapR luxO* double-SNP strain due to a reduction in the *hapR* message; *per contra*, at high cell densities (7.5 h), we hypothesized a decrease in TcpA and CT production due to the derepression of the *hapR* message. However, the *hapR luxO* double-SNP strain exhibited a decrease in ToxT production (0.8-fold) and no statistically significant change in TcpA production, and an increase in CT production (1.6-fold) resulted.

VieA contains a domain that binds and degrades the prokaryotic secondary-messenger c-diGMP, and at high levels, c-diGMP inhibits expression of both the *ctxAB* and *toxT* operons (35, 38). The degradation of this secondary messenger is required for full activation of the *ctxAB* operon. When the nonsynonymous SNP in *vieA* (C_235_T, residue L_79_F) was introduced into N16961, there was no statistically significant change in CT or ToxT production, but TcpA production decreased (0.7-fold), suggesting that the SNP in *vieA* alters VieA activity toward the *tcp* operon. This altered VieA activity is currently under further investigation.

Our data show that CT, TcpA, and ToxT levels observed in hypervirulent MQ1795 were not mirrored in any of the single-SNP strains (Fig. 1 to 3). TcpA production profiles revealed that multiple double- and triple-SNP combinations (Fig. 2) produced TcpA levels similar to that of MQ1795. Only the *hns luxO vieA* triple-SNP combination strain produced ToxT levels comparable to that of MQ1795 (Fig. 3). The greatest increases in TcpA and ToxT productions were observed in the *hns luxO vieA* triple-SNP combination strain (Fig. 2 to 3). CT production profiles revealed that the quadruple-SNP combination strain produced the greatest increase in CT but did not mirror levels produced by MQ1795 (Fig. 1). The addition of functional *hapR* to *hns luxO vieA* in the quadruple-SNP strain produced the greatest increase in CT production (Fig. 1); however, the *hns luxO vieA* SNP strain exhibited the highest increase in TcpA (Fig. 2) and ToxT (Fig. 3) production.

The data presented here are limited to examining CT, TcpA, and ToxT production profiles, and future directions involve investigating the effects that each SNP or SNP combination has on other phenotypic traits, i.e., motility, biofilm formation, hemolytic capabilities, and other metabolic activities. In addition to negatively affecting CT and TCP production, H-NS has previously been shown to repress exopolysaccharide gene (*vps*) expression and biofilm formation (44). It was reported that repression of HapR, via a LuxO mutant, also allowed for the enhanced expression of hemagglutinin (HA)/protease and biofilm production (45). Additionally, a *vieA* deletion resulted in the buildup of c-diGMP, which has been reported to increase biofilm formation and decrease motility (39). Further characterization of these SNPs on a phenotypic level in conjunction with the data presented here will help to further elucidate the exact mechanisms allowing for increased virulence capabilities observed in clinical isolates of *V. cholerae* and severe cases of cholera.

## MATERIALS AND METHODS

### Bacterial strains and general growth conditions.

All strains used in this study are listed in Table 2. Regulatory SNPs identified in MQ1795 (*hapR*, *hns*, *luxO*, *vieA*) (9) were introduced into the El Tor reference strain N16961 using an established allelic-exchange protocol (46) and the primers listed in Table 3. SNPs and SNP combinations were verified by PCR using the primers also listed in Table 3. Strains were cultured under standard growth conditions in Luria-Bertani broth at 37°C overnight (16 h) with aeration. El Tor virulence gene expression growth conditions were as previously described (47). Briefly, 10 ml of AKI medium containing 0.03% (wt/vol) sodium bicarbonate was inoculated with a single colony and grown for 3.5 h at 37°C without aeration. Seven milliliters of culture was removed, and the whole-cell extract (WCE) was processed for ToxT analysis. The remaining 3 ml of culture was incubated for an additional 4 h at 37°C with aeration. The WCE was processed for TcpA analysis, and the cell-free culture supernatant was prepared for CT analysis.

**TABLE 2:** *Escherichia coli* and *Vibrio cholerae* strains used in this study^a^

Strain	Description	Reference or source
*Escherichia coli*		
S17-λpir	*recA thi pro* hsdR*^−^* M^+^ [RP4-2-Tc::Mu::Km^r^ Tn*7*] (λ*pir*) Tmp^r^ Str^r^	de Lorenzo and Timmis (49)
*Vibrio cholerae*		
O395	Classical wild type, Str^r^	Son et al. (9)
C6706	El Tor wild type, Str^r^	Son et al. (9)
N16961	El Tor reference strain, Str^r^	Son et al. (9)
MQ1795	El Tor variant, clinical isolate	Nair et al. (27)
MS0005	Classical O395Δ*toxT*, St^r^	Son et al. (9)
MS0014	Classical O395Δ*tcpA*, St^r^	Kirn et al. (50)
MS0235	El Tor N16961 hapR* St^r^	This study
MS0245	El Tor N16961 hns* St^r^	This study
MS0230	El Tor N16961 luxO* St^r^	This study
MS0240	El Tor N16961 vieA* St^r^	This study
MS0256	El Tor N16961 hapR* hns* St^r^	This study
MS0254	El Tor N16961 hapR* luxO* St^r^	This study
MS0251	El Tor N16961 hapR* vieA* St^r^	This study
MS0285	El Tor N16961 hns* luxO* St^r^	This study
MS0281	El Tor N16961 hns* vieA* St^r^	This study
MS0287	El Tor N16961 luxO* vieA* St^r^	This study
MS0279	El Tor N16961 hapR* hns* luxO* St^r^	This study
MS0295	El Tor N16961 hapR* hns* vieA* St^r^	This study
MS0297	El Tor N16961 hapR* luxO* vieA* St^r^	This study
MS0299	El Tor N16961 hns* luxO* vieA* St^r^	This study
MS0282	El Tor N16961 hapR* hns* luxO* vieA* St^r^	This study

^a^ S17-λ*pir* was used to introduce all possible SNPs and SNP combinations into the El Tor reference strain N16961, using a previously described allelic-exchange protocol (46). SNPs introduced are indicated by asterisks. Tmp^r^ represents trimethoprim resistance, and Str^r^ represents streptomycin resistance.

**TABLE 3:** Primers used in this study to introduce SNPs and verify sequencing

Oligonucleotide no.	Oligonucleotide name	Sequence (5′–3′)^a^	Purpose
0159	ETV-*hapR*-Forward	GATCG*GAATTC*CCATTTCCTACTTGAAGCTGTAGCGGTG*TTGGCAG*	Allelic exchange
0160	ETV-*hapR*-SapI-Reverse	GATCGGCTCTTCACGTCAGTACTCCAACTTCTTGACCG*ATCACATCG*	Allelic exchange
0161	ETV-*hapR*-SapI-Forward	GATCGGCTCTTCAACGAACCACAAAATTCAGCACATCGTC*AACCAAGTCTTC*	Allelic exchange
0162	ETV-*hapR*-Reverse	GATCG*AGATCT*CAGTATCGCTGACTTTGGTGGCGCG*TATAGTACC*	Allelic exchange
0163	ETV-*luxO*-Forward	GATCG*GAATTC*GCTGGATATTGATATCAATATCGTGGG*TACCGG*	Allelic exchange
0164	ETV-*luxO*-SapI-Reverse	GATCGGCTCTTCACGCCTTGACGCTCAGTCGCCGCCCCAG*TAAAAGC*	Allelic exchange
0165	ETV-*luxO*-SapI-Forward	GATCGGCTCTTCAGCGTGGCAGAAGCGGCTGATGGGGG*AACCCTCTTTTTGG*	Allelic exchange
0166	ETV-*luxO*-Reverse	GATCG*AGATCT*GCTTGTTCAATGGCTTGTTTTTCGGTC*ATCCACAGC*	Allelic exchange
0167	ETV-*hns*-Forward	GATCG*GAATTC*GGTTTATTTATGGCGGGTTACACCGAAG*ATTCCG*	Allelic exchange
0168	ETV-*hns*-SapI-Reverse	GATCGGCTCTTCAGTGAAGAAAAAACTTGGACAGGCCAAG*GCCGTACTCC*	Allelic exchange
0169	ETV-*hns*-SapI-Forward	GATCGGCTCTTCACACTGTTGGTGTCGATATACTTGTAT*TTTGCAGGGCGAGG*	Allelic exchange
0170	ETV-*hns*-Reverse	GATCG*AGATCT*CAATCATTTCATTGATACTTATTTCATAA*AAACACC*	Allelic exchange
0171	ETV-*vieA*-Forward	GATCG*GAATTC*GGTCGGGGTTCCGTTGTACACCTCATGC*TCCGTC*	Allelic exchange
0172	ETV-*vieA*-SapI-Reverse	GATCGGCTCTTCATCAGCGCTGTGGAAGATACGATTCTTG*AGTTAAC*	Allelic exchange
0173	ETV-*vieA*-SapI-Forward	GATCGGCTCTTCATGAATATCACCACACCTAGCTTAGGTG*CCTGTAAACTCAG*	Allelic exchange
0174	ETV-*vieA*-Reverse	GATCG*AGATCT*CAGTCGTTATCTCACAACACAATGGGTCG*CACTGCC*	Allelic exchange
0212	*hapR*-Upstream	GCATTGTATAAATGGGGCTTGGAGAATTTAGGCG	Sequencing
0213	*hapR*-Downstream	GCTCAGTGATCTGTTGACCTAATTCGCGAATGCG	Sequencing
0214	*hns*-Upstream	GCATTAGCTTTAACAGGAGAAAGCGATCCGCTCGC	Sequencing
0215	*hns*-Downstream	GCAACTAGGTTCCAGTGAGAAAAACAAGTGCCACAGC	Sequencing
0216	*luxO*-Upstream	GGCTAGGCTATGCAACATAATCAATCTTTGCAG	Sequencing
0217	*luxO*-Downstream	CCAAGTTTGCAGCTTGCGATAGATGGTTGACGG	Sequencing
0218	*vieA*-Upstream	GCAGTTGAGCCAGTTGACCTAATGACGCGTAACC	Sequencing
0219	*vieA*-Downstream	GCGGAACAAGCTCTGCAAGCGGGTATGGATAAGG	Sequencing

^a^ Italics represent regions of enzyme restriction sites.

### CT production assay.

GM_1_ ganglioside enzyme-linked immunosorbent cholera toxin (CT) assays were performed on 7.5-h culture supernatants for all *V. cholerae* strains as previously described (48). Threefold dilutions of a CT standard (List Biological Laboratories, CA, USA), uninoculated media, El Tor N16961, clinical isolate MQ1795, O395Δ*toxT*, and all strains containing SNPs were prepared in GM_1_ ganglioside (Sigma-Aldrich, MO, USA)-coated 96-well plates. Samples were probed with anti-CT_B_ (List Biological Laboratories, CA, USA) to determine the total nanograms of CT produced per milliliter of culture per optical density at 600 nm (OD_600_), and fold changes in CT production from that of N16961 were calculated.

### Immunoblot assays for ToxT and TcpA.

WCE were prepared from all the cultures grown under AKI-inducing conditions for 3.5 h (for ToxT) or 7.5 h (for TcpA) at 37°C, as described above. Total protein concentrations were prepared to a final concentration of 0.5 µg/µl. Totals of 12 µg and 3 µg total protein were analyzed for ToxT and TcpA, respectively. For ToxT analysis, samples were run on 14% Tris-glycine polyacrylamide gels (Invitrogen, Carlsbad, CA, USA) and 16% Tris-glycine polyacrylamide gels for TcpA. Samples were then transferred to nitrocellulose membranes, probed with anti-ToxT or anti-TcpA (both generous gifts from Ronald K. Taylor), and visualized using the enhanced-chemiluminescence (ECL) detection system (GE Healthcare, Little Chalfont, Buckinghamshire, United Kingdom). The band intensities, relative to those of N16961, were determined by densitometry using Image Lab 4.0.1 software (Bio-Rad Laboratories, USA).

### Statistical analysis.

Fold changes in CT were determined relative to the CT level of the El Tor reference strain N16961. TcpA and ToxT immunoblots were normalized using nonspecific bands from each blot, and fold changes from N16961 were determined. Outliers were removed after calculating the upper (3rd quartile plus the interquartile range times 1.5) and lower (1st quartile minus the interquartile range times 1.5) boundaries of relative fold changes across respective strains prior to determination of statistical significance. CT assays were conducted in 10 independent experimental replicates, with each replicate representing an independent culturing event for each trial. Across all CT assays, 10 outliers were observed in 6 strains (1 outlier in the *hapR luxO*, *hapR hns vieA*, and *hns luxO vieA* strains, 2 outliers in the O395Δ*toxT* and *hapR* strains, and 3 outliers in the *hns* strain). Similarly, analysis of the results of five TcpA and seven ToxT independent experimental replicates was also conducted, with each replicate representing an independent culturing event for each trial. We identified 11 outliers during TcpA analysis across 9 strains (1 outlier in the C6706, O395Δ*toxT*, O395Δ*tcpA*, *hns*, *vieA*, *hapR vieA*, and *hapR luxO vieA* strains and 2 outliers in the *hapR hns* and *hapR hns vieA* strains). Analogously, 11 outliers were identified during ToxT analysis across 10 strains (1 outlier in the O395Δ*toxT*, O395Δ*tcpA*, *hapR*, *luxO*, *vieA*, *hapR luxO*, *hns vieA*, *hapR luxO vieA*, and *hns luxO vieA* strains and two outliers in the *hns luxO* strain). A final two-tailed Student *t* test was conducted for CT and both immunoblot assays, and statistical significance was determined at a *P* value of ≤0.05.
